# Editorial: Selenium in soil-plant-animal systems and its essential role for human health

**DOI:** 10.3389/fpls.2023.1237646

**Published:** 2023-07-27

**Authors:** Gary S. Banuelos, Zhi-Qing Lin, Joel Caton

**Affiliations:** ^1^ United States Department of Agriculture, Agricultural Research Service, Parlier, CA, United States; ^2^ Department of Environmental Sciences, Southern Illinois University, Edwardsville, IL, United States; ^3^ Department of Biological Sciences, Southern Illinois University, Edwardsville, IL, United States; ^4^ Department of Animal Sciences, North Dakota State University, Fargo, ND, United States

**Keywords:** selenium, soil, plant, animal, biofortification, human health

With approximate 1 billion people facing some degrees of selenium (Se) deficiency worldwide, it is imperative that the Se community work together and share the latest knowledge on various inter-related aspects of Se in supporting and protecting animal, human, and ecosystem health. In collaboration with Frontiers of Plant Science, Frontiers in Nutrition, and Frontiers in Veterinary Science, this Research Topic entitled *Selenium in soil-plant-animal systems and its essential role for human health* published original research reports and critical reviews representing different but interrelated research disciplines involving physiochemical and biological behaviors of Se within the larger foundation topics of agricultural soil, bioavailability, plant uptake, physiological responses, genetics, molecular biology, microbial communities, Se-biofortification strategies, and animal health.

Selenium is unevenly distributed in the soil, which has consequently resulted in soil Se deficiency issues and further low Se dietary intake in many parts of the world. To increase Se intake by consumption of Se-enriched plant- and animal-derived food products, we need to better understand and identify those effective strategies for Se delivery though agricultural production systems in different geographical regions. Enhancing bioavailable Se in soil will not only increase Se accumulation in crops but also result in the accumulation of specific bioactive Se compounds in food products. Importantly, the true value of successfully increasing Se concentrations in plant- and animal-based products could be highly determined by the fractionation and the speciation of Se, such as seleno amino acids or selenoproteins in Se-biofortified food products. The different Se compounds accumulated in plant tissues would further determine their bio-accessibility and the absorption of Se through human digestion systems. Similarly, animal health and reproduction are also very much dependent on the bioavailability and the absorption of Se from feeding materials. The adequate intake of Se from the feeding materials or using supplementary Se significantly affect animal’s vital physiological functions that are related to reproduction or pregnancy health, and their auto-immune functions.

In this Research Topic, 15 high-quality research papers that addressed various topics or faces of Se research, ranging from the biogeochemical cycling of Se to cellular and molecular processes that elucidate mechanistic functions of Se in human and animal health. Inorganic and organic Se transformations and physio-chemical properties of soils could all ultimately determine soil Se bioavailability for plant uptake and accumulation. Both the concentration and the speciation of Se in soil could affect the Se content and the Se status of crops, particularly in edible plant materials. Recent studies demonstrated that biofortification as an agronomic-based strategy can be utilized to mitigate a low transfer of Se and other nutrients from soil into the food chain and produce Se-enriched food products, which helps increasing dietary Se intake throughout Se-deficient susceptible regions of the world.

Agronomic Se biofortification has been commonly practiced by adding Se-amended fertilizers to the soil. In Brazil, soybean is a potential major crop for biofortification. In “*Se biofortification of soybean genotypes in a tropical soil via Se-enriched phosphate fertilizers*”, Silva et al. showed that the application of Se-amended phosphate fertilizers could be an effective method to deliver Se to the crop. Adding Se to the commonly used fertilizers could also be challenging due to the soil Se concentration baselines, soil types, redox protentional, pH, and soil microbial or invertebrate communities. Song et al. indicated in “*Selenium effect threshold for soil nematodes under rice biofortification”* that, with the application of selenite for rice biofortification, higher concentrations of soil Se can negatively affect soil nematodes, suggesting that the presence of soil nematodes could be used as an effective bioindicator for the soil environmental changes related to Se content. In addition to the uptake of inorganic Se, plants can also absorb Se *via* organic Se application, as shown in “*Uptake and translocation mechanisms of different forms of organic Se in rice”* by Wang, Q. et al. This rice study provides important insights into the mechanisms underlying the uptake and translocation of organic Se, especially selenomethionine (SeMet), in plants. As an alternative to soil applied Se, foliar application has been used to apply Se to plants. Schiavon et al. indicated that *“Foliar Se fertilization alters the content of dietary phytochemicals in two rocket species*,” while Wang, M. et al. further outlined the differences between soil and foliar Se applications in a paper entitled “*Soil and foliar Se application: Impact on accumulation, speciation, and bio accessibility of Se in wheat*”. In addition to foliar Se application, Malka et al. evaluated potential interactions between Se and Zn in foliar application, and indicated that “*Separate foliar sodium selenate and zinc oxide application enhances Se but not Zn accumulation in pea seeds”.* Foliar application of Se may additionally influence plant metabolism, as well as increasing Se content in plant tissues, as shown by de Sousa et al. in “*Selenium enhances chilling stress tolerance in coffee species by modulating nutrient, carbohydrates, and amino acids context”*, and demonstrated that foliar Se application improved coffee plants’ ability to tolerate chilling stress.

To produce Se-enriched agricultural products, the biofortification strategy can also be practiced in regions where there are naturally high levels of Se in the soil and/or in irrigation waters, as demonstrated by Banuelos et al. in “*Salsola soda (agretti) as a Se biofortification crop grown under high saline and boron conditions*.” Under field conditions Se-biofortified *Salsola soda* was produced with poor quality waters containing high levels of Se. Careful attention must, however, be paid in regions where Se-biofortified crops are grown in naturally Se-rich soils or with poor quality waters because of the potential presence of toxic metals in the environment. In “*Prediction models for monitoring Se and its associated heavy-metal accumulation in four kinds of acro-foods in seleniferous area”*, Jiao et al. demonstrated that models can be used to effectively predict toxic metal accumulation in Se-enriched foods in those concerned regions.

Se-enriched food products can increase Se intake and promote human health with absorption of plant tissue containing different Se compounds including seleno-amino acids. Earlier studies have clearly demonstrated the important role of Se in plant and animal physiological processes and functions. Hu et al. reviewed the importance of “S*eleno-amino acids in vegetables”*, a review of their forms and metabolism and thereby affect protein structures, functional properties and antioxidant capacity in newly-germinated Se-enriched soybeans. Relatedly, Huang et al. also reported in “*Selenium biofortification of soybean sprouts: effects of Se enrichment on proteins, protein structure, and functional properties”* that Se-biofortified seeds also contain proteins whose quality has also been influenced by Se content. In addition, Li et al. evaluated “*The use of selenium for controlling plant fungal diseases and insect pests*”, indicating that Se improves the plant resistance to fungal diseases.

Excessive low or high Se in soil and consequently Se concentrations in animal feeds can pose health and reproduction risks for animals. Animal-based food products for human consumption are an excellent source of dietary Se intake for the human population. Thus, safely providing Se biofortified feed materials to animals would result in increased Se concentrations in animal-based food products for humans. Hall et al. discussed Se biofortification through forages raised for livestock feed in “*Impact of selenium biofortification on production characteristics of forages grown following standard management practices in Oregon*,” demonstrating that foliar selenate treatment increased forage Se concentrations in a dose-dependent manner, and that coupling Se amendment with standard fertilization practices promoted forage growth and forage Se concentrations. In cases of low soil Se, providing sulfur fertilization could reduce forage Se and potentially alter Se supply to livestock consuming those forages.

A major determinant of livestock production, health, and well-being is effective and efficient reproductive process that lead to healthy offspring. Dahlen et al. reviewed the role of Se in male and female reproductive process and the impacts of maternal dietary Se on offspring outcomes in ruminants in their paper “*Selenium supplementation and pregnancy outcomes*.” The scientific evidence indicates that Se plays a major role in both male and female reproductive processes and, therefore, as a micronutrient, Se is instrumental to ensure successful animal reproductive efficiency. Increasing the maternal supply of Se alters offspring outcomes in ways that are typical of developmental programming; thereby implying that Se supply to the mother can have significant effects into the next generation of livestock. In animals, mitochondrial function is essential to bioenergetics and consequently life functions. Clearly, the role of Se in antioxidants plays a role in normal cellular metabolism and consequently whole animal health, production, and wellbeing. In addition, Se appears to have a role in mitochondrial function besides through antioxidants. Wesolowski et al. reviewed the non-antioxidant the roles of Se in mitochondrial function in “*Beyond antioxidants: Selenium and skeletal muscle mitochondria*.” The review paper demonstrates our emerging understanding of the role of Se in skeletal muscle mitochondrial function beyond the traditional constructs of antioxidants, and further highlights the importance of a greater understanding of Se in mitochondrial function and energetics.

Selenium is one of the most influential natural-occurring micronutrient elements for living systems. Recognizing selenium’s impact on a multitude of processes in nature requires multi-disciplinary research on Se absorption, chemical transformation, and biochemical and physiological metabolisms in soil-plant-animal systems that can help us develop and implement effective strategies to mitigate public health impacts or concerns of Se deficiencies in the world. In this Research Topic, with different contributions from original research to critical reviews, some of the most influential researchers have provided their latest research findings and demonstrated significant advances in the field concerned with Se in food chains and its effects on human and animal health.

## Author contributions

All authors (GB, Z-QL, and JC) contributed to the article through writing, reviewing and editing and have approved the submitted version.

